# Research elevation of bank lending and technological innovation in the excess liquidity countries

**DOI:** 10.1016/j.heliyon.2024.e33462

**Published:** 2024-06-22

**Authors:** Agus Salim, Suripto, Dini Yuniarti, Ignatius Abasimi, Nurul Azizah Az Zakiyyah, Indanazulfa Qurrota A'yun

**Affiliations:** a Department of Development Economics, Faculty of Economics and Business, Universitas Ahmad Dahlan, Special Region of Yogyakarta, Indonesia; b Department of Urban and Regional Planning, School of Geographical Science, Northeast Normal University, Changchun, Jilin, PR China

**Keywords:** Bank lending, Technological innovation, Excess liquidity, Bibliometrics analysis, Research direction

## Abstract

This study examines the intellectual framework research in bank lending and technological innovation relationships in countries with high banking system liquidity. This study employs bibliometrics with R-studio tools and procedures to analyze documents regarding productions, collaborations, keyword occurrences, conceptual structure, and density and centrality occurrence's network. Combining data from the Web of Science and Scopus databases, this study obtained 939 documents from 527 sources with a significant opportunity for further elevation through combination with other themes. The development analysis based on the most related countries indicates that researchers from other countries have also conducted studies identified as having significant banking liquidity. Topic development and thematic evolution show that research on the role of bank lending on technological innovation evolves to environmental issues, with *green credit* as the most recent and emerging elaboration. For further direction, keywords in *investment* clusters can help elevate *education, commerce,* and *impact* clusters by combining them with research on *government taxation*, *credit provision*, *sustainable development*, and *emission control* themes.

## 1 Introduction

Technological innovation is essential in improving economic growth by lowering manufacturing costs and enabling higher output. Therefore, the presence of technological innovation would increase efficiency [[1], [2], [3], [4], [5], [6]]. In other words, countries that have more innovation would produce more productivity. The role of innovation as a crucial aspect in improving the whole life of the economy become another focus of researchers to find the key determinants affecting innovation from various areas of study. The study of Raghupathi & Raghupathi [2] shows that countries with lower GDP have greater dependence on foreign collaboration of innovation. It means higher GDP produces more prosperous innovation independently. Wen & Usman [7] stated that exchange rate depreciation improves national innovation through trade openness. Ebersberger [8] and Tang et al. [9] suggest a government subsidy to enhance innovation by alleviating financial constraints, improving labor's creativity, and spurring technological innovation. Another macroeconomic indicator, inflation, negatively affects innovation created by firms with a higher dependence on liquidity [10]. Therefore, in the macroeconomy aspect, the financial sector's presence must be addressed as a vital instrument in providing liquidity funding for producing innovation.

The financial sector is growing in facilitating technological innovation by providing readily available funds for innovation projects [[11], [12], [13]]. Schumpeter's [14] earlier work explained that sufficient loans from financial institutions to companies promote the creation of innovations. Financial backing significantly impacts enterprises that heavily rely on external funding and engage in complex goods, systems, and technological applications. Economic expansion and institutions, along with their lending practices, are closely linked to fostering innovation. Their availability is connected to corporate processes of mobility and innovation generation [15,16]. Financial sector borrowing offers benefits for the allocation of resources and risk management. Previous studies analyze how financial loans affect invention, such as the type of financial development in various markets such as the stock market [17], real estate market in housing prices [18], and insurance in managerial [19]. They revolve around how the advancements in financial marketplaces bolster the technical innovation of the organization.

Bank financing in the financial sector should positively influence a firm's technological innovation [20]. Data provided by International Financial Statistics (IFS) of the International Monetary Fund (IMF) shows that Canada, China, Hong Kong, Japan, and Luxembourg have consistently ranked among the top 5 over 30 countries with the highest levels of excess liquidity in their banking sectors over two decades [21]. Luxembourg has the highest average liquid liability from 2002 to 2021, around 729.97 percent of GDP. It is followed by Hong Kong, Japan, China, and Canada, at around 335.32 percent, 216.63 percent, 179.08 percent, and 170.64 percent of GDP, respectively. Theoretically, excess liquidity countries may offer significant financial assistance to support their economic growth [[22], [23], [24], [25]]. In particular, they can help companies by improving business innovation, boosting infrastructure investment, and expanding R&D personnel.

Recent studies provided a more comprehensive role of financial development in terms of banking liquidity in various aspects, such as the role of credit policy in reducing the pollution effect [26], the role of financial technology (fintech) lending on innovation [27], and the role of financial development on the enhancement of export city level [28]. Regarding the ecological aspect, the study of S. Liu et al. [26] promotes that the green credit system has a more significant impact on environmentally friendly innovations and the success of high-polluting and energy-intensive state-owned firms with inadequate market strength. Furthermore, the green credit policy can alter the credit financing limitations and R&D investment allocation to impact the environmentally friendly innovation success of high-polluting and energy-intensive companies. Ding et al. [27] investigate and analyze the influence of fintech advancements on innovation and pinpoint the underlying economic mechanisms that drive this phenomenon. They concluded that fintech advancements encourage business lending and R&D spending because internet credit heightens competition for bank loans. The study of Pan et al. [28] shows that digital inclusive financing may greatly enhance export upgrading, and cities with smaller sizes, lower salaries, higher human capital levels, and better geographical advantages have more positive benefits of digital inclusive financing in enhancing export upgrading.

In contrast, the research on the function of bank lending in supporting innovation is still a subject of discussion due to theoretically uncertain conclusions. Hsu et al. [29] researched how the growth of equity and credit markets affects innovation. They discovered that the growth of credit markets hinders invention in firms that rely more on external funding and have higher levels of technological intensity. N. Lee et al. [30] discovered that creative enterprises had challenges securing bank finance. Research conducted by Beck et al. [31] found that liquidity creation boosts tangible investment but does not affect intangible innovation across nations and industries that rely more on debt finance. Sharma et al. [32] propose that many high-tech enterprises struggle to secure bank loans because of unequal access to information and the absence of assets suitable for collateral. Therefore, creative companies with intangible assets do not receive financial assistance from banks. Hence, regarding the contrast debate aformentioned above, there is an urgent need to provide novel insights into the performance, topic elaboration, thematic evolution, and further research elevation in bank lending and technological innovation.

This study addresses the existing knowledge gap and answers the research inquiries, as follows:

1. What is the scientific performance of research in bank lending and technological innovation in the top 5 countries with the most liquid banking systems?
2. What is the research development of bank lending and technological innovation?
3. What is the research's current focus of bank lending and technological innovation?
4. How the research theme of bank lending and technological innovation in the top 5 liquid banking countries evolved over the global financial crisis of 2008/2009 and during the COVID-19 pandemic?

Ultimately, this work offers an in-depth examination and suggests avenues for further research elevation in bank lending and technological innovation.

## 2 Data Collection and method

### 2.1 Data

**Fig. 1:**
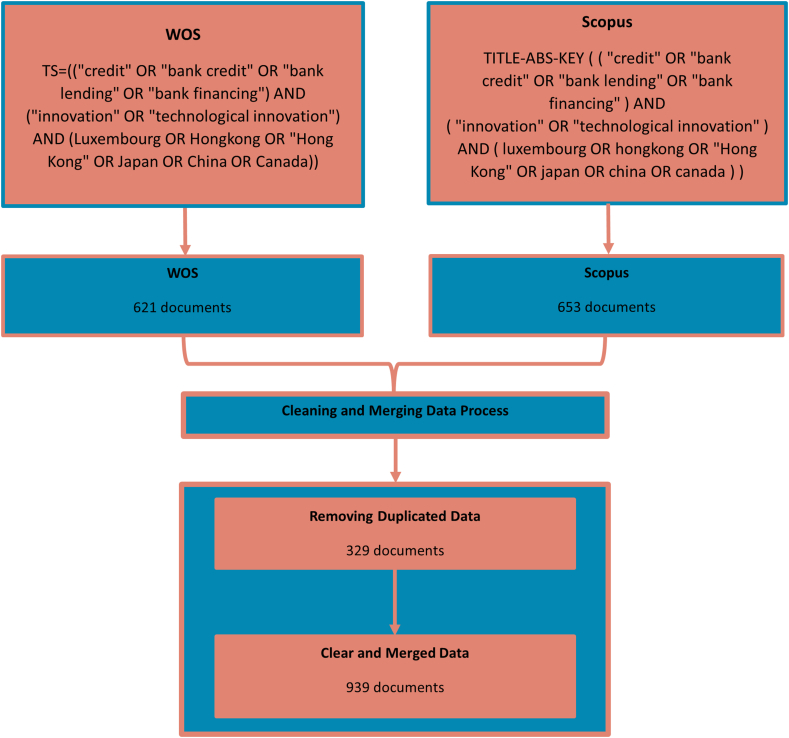
Data collection and matching process.

This study gathered manuscripts containing keywords related to "bank lending" and "technological innovation" topics, including titles, abstracts, and keywords, from the Web of Science (WOS) and Scopus databases. This study includes the names of the top 5 most liquid banking countries (Luxembourg, Hong Kong, Japan, China, and Canada) to discuss particular concerns. By eliminating 329 duplications, this study combined the data and obtained 939 documents from 3171 Journals. The tool assisted in this research used to analyze 621 documents published in WOS from the Web of Knowledge database: https://www.webofscience.com/wos/alldb/basic-search and 653 documents from Scopus https://www.scopus.com/. To provide a complete source within the topics of bank lending and firm's technological innovation, this study does not apply any limitations provided by both sources. Fig. 1 provides access to the matching process and query string, including research keywords in both sources.

We utilized C. Wang et al.'s [33] strategy for the data cleaning method, focusing on integrating author-keywords, country, and the whole data, as follows:

1. Merging the synonym of keywords of bank lending, such as "credit" OR "bank credit" OR "bank lending" OR "bank financing";
2. Merging the synonym of keywords of technological innovation, such as "innovation" OR "technological innovation";
3. Merging the synonym of keywords of country-specific, such as Luxembourg OR Hongkong OR "Hong Kong" OR Japan OR China OR Canada;
4. At the same time, the Author-keywords (DE) field is absent from certain publications. The approach used with Keywords Plus (ID) was meant to close this gap. If ID information is absent, DE is manually added using the title;
5. After obtaining the data from Scopus and Web of Science (WOS), it is imported into R-Studio using the following code:
  • scopus_data < -convert2df("scopus.bib",dbsource = "scopus",format = "bibtex") for data obtained from Scopus; and
  • web_data < -convert2df("WOS.txt") for data obtained from Web of Science.
6. Merge the data from both sources using the code: combined < -mergeDbSources(web_data, scopus_data,remove.duplicated = T); and
7. To simplify uploading data and analysis into biblioshiny or bibliometrix, export the merged data in Excel using the code write.xlsx(combined,"combineddata.xlsx").

### 2.2 Tools of analysis

This study utilizes the R-package blibliometrix analytical technique to examine bibliometrics designed by Aria & Cuccurullo [34]. The Bibliometix program in the R language is highly adaptable and frequently updated, allowing for seamless integration with other statistical R packages. Thus, it is highly advantageous in bibliometrics, characterized by its dynamic nature. Moreover, Corbet et al. (2019) elucidate that the purpose of employing the R package bibliometrix is to uncover the primary patterns by visually representing the intellectual framework of a specific subject area. Therefore, it is beneficial to help us answer the research question and elevate future research development in bank lending and technological innovation.

### 2.3 Scientometrics method

The number of papers, sources, authors, documents, and conceptual structure is used in four stages to analyze the bibliometrics to help answer the research questions and provide future research direction.

#### 2.3.1 Overview analysis

Overview analysis explains the central insight and general information of the data collected. The table is a starting point for understanding the rest of this article's analysis. It includes publications and average total citations [34]. This study used an overview analysis provided by *bibliometrix* to help explain the scientific production of research in bank lending and technological innovation, such as primary information (timespan, growth, and age), authorship, and documents. This analysis is employed to answer the research question of the scientific performance of research in bank lending and technological innovation in the top 5 countries with the most liquid banking systems.

#### 2.3.2 Most relevant country analysis

This study provides the most relevant countries by corresponding authors. This study quantifies the scientific output of countries in terms of publications and the extent of international collaboration in stylometry research, focusing on the country of the corresponding author [35]. The result of this analysis is used to analyze the development of research authorship collaboration. Furthermore, this analysis helps review whether the research is broadly analyzed.

#### 2.3.3 Trending topic analysis

This analysis gives further insight into the trending topics regarding keyword occurrences in intelligent learning literature over the years [36]. To visualize the recent trend of topical development dynamic, the study first calculates the median year for each keyword since 2011, and we choose the top 3 keywords in each year with a minimum frequency of 3. The length of the bar presents the first and last point of mentioning the keyword based on the frequency of collected keywords [37].

#### 2.3.4 Thematic evolution analysis

Research thematic evolution is part of conceptual structure in bibliometrics analysis [38]. It performs a thematic evolution analysis based on co-word network analysis and clustering provided by Cobo et al. [39]. To answer the research question of how the research theme evolution of bank lending and technological innovation in the top 5 liquid banking countries before and after the global financial crisis of 2008/2009 and during the COVID-19 pandemic, this study includes thematic movements divided into before and during GFC (1984–2010), post-GCF and pandemic covid-19 (2011–2021), and post-pandemic covid-19 (2022-early 2024).

#### 2.3.5 Thematic map analysis

Ultimately, this study employs authors’ keywords and their interconnections to obtain themes to provide further research direction by analyzing research thematic maps based on the density and centrality of co-occurrence networks. This study employs a thematic map in two dimensions—density and centrality—defined by Callon et al. [40]. Co-word analysis of the thematic map draws clusters of keywords. They are considered themes whose density and centrality can be used in classifying themes and mapping in a two-dimensional diagram [34].

## 3 Result and discussion

### 3.1 Scientific performance: an overview analysis

The analysis answers the first question of the research development of bank lending and technological innovation in the top 5 countries with the most liquid banking systems. Table 1 presents the primary descriptive statistics from a database of documents encompassing published manuscripts from 1984 to early publications in 2024. This study gathered 939 documents from 527 sources, and the collection had 21,858 cited references. All the manuscripts are written by 1431 authors, mainly dominated by research in articles with 598 documents. Moreover, the yearly growth rate of this study is 1.02, and the average age is 4.57 years. The data suggests that more research publications on bank lending and technological innovation are needed. It presents a significant opportunity for further elevation through combination with other themes.

**Table 1:** Main information of the dataset.

Description	Results	Description	Results
MAIN INFORMATION ABOUT DATA		DOCUMENT TYPES	
Timespan	1984:2024	article	598
Sources (Journals, Books, etc)	527	article; article	2
Documents	939	article book chapter	1
Annual Growth Rate %	1.02	article conference paper	1
Document Average Age	4.57	article conference review	1
Average citations per doc	11.89	article; early access	49
References	21858	article; proceedings paper	2
DOCUMENT CONTENTS		book	10
Keywords Plus (ID)	2465	book chapter	36
Author's Keywords (DE)	2346	conference paper	70
AUTHORS		conference paper review	1
Authors	1431	conference review	17
Authors of single-authored docs	144	editorial	1
AUTHORS COLLABORATION		editorial material	2
Single-authored docs	182	erratum	9
Co-Authors per Doc	2.86	proceedings paper	110
International co-authorships %	11.93	retracted	4
		review	21
		short survey	4

**Fig. 2:**
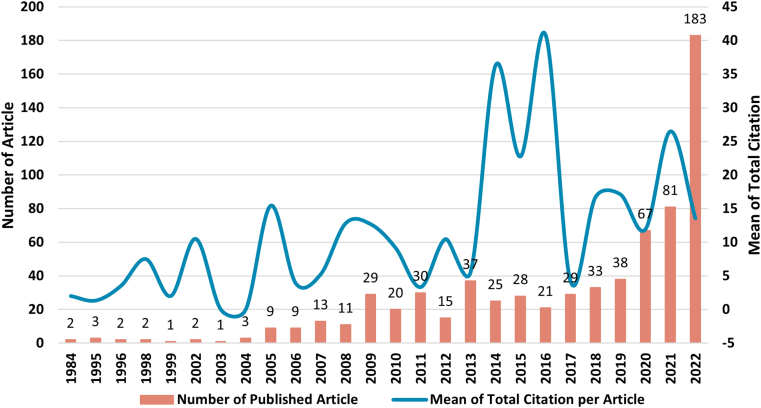
Annual production and total citation per year.

Moreover, Fig. 2 illustrates the upward trajectory of annual research publications and the mean of total citations. The number of articles produced has increased from only two in 1984 to 183 in the most recent published manuscript. The average number of citations steadily rises, reaching its highest point at 40.86 in 2016. Table 2 shows publications and citations in detail. Guo & Liang [41], who analyzed the current issue of financial innovation in the banking system, were nominated as the most cited documents with 552 total citations. In terms of the role of bank lending in reducing environmental issues, C. H. Yu et al. [42] gained 352 citations after being published in 2021. Other research-related disciplines, such as human capital [43] and government policies [44], gained 216 and 276 citations, respectively. The statement indicates that although the production growth is relatively low, a significant interest in conducting research has a strong demand for referring to an article analyzing bank lending and technological innovation. The details of movements are analyzed in the following part.

**Table 2:** Top 10 cited documents.

No.	Document	Total Citations	TC per Year	Normalized TC
1	Guo & Liang [41]. “Blockchain application and outlook in the banking industry”	552	69.00	13.51
2	Yu et al. [42]. “Demand for green finance: Resolving financing constraints on green innovation in China”	352	117.33	13.29
3	Cull et al. [44]. “Government connections and financial constraints: Evidence from a large representative sample of Chinese firms”	276	30.67	12.13
4	Hu et al. [45]. “Can the green credit policy stimulate green innovation in heavily polluting enterprises? Evidence from a quasi-natural experiment in China”	259	86.33	9.78
5	J. Hu et al. [43]. “Leader humility and team creativity: The role of team information sharing, psychological safety, and power distance”	216	36.00	12.94
6	Cao et al. [46]. “Digital finance, green technological innovation and energy-environmental performance: Evidence from China's regional economies”	209	69.67	7.89
7	Guan & Yam [47]. “Effects of government financial incentives on firms' innovation performance in China: Evidences from Beijing in the 1990s”	207	23.00	9.10
8	Irfan et al. [48]. “Influence mechanism between green finance and green innovation: Exploring regional policy intervention effects in China”	203	101.50	14.97
9	Yao et al. [49]. “Green credit policy and firm performance: What we learn from China”	160	53.33	6.04
10	Chen et al. [50]. “Knowledge management and innovativeness: The role of organizational climate and structure”	156	11.14	17.05

### 3.2 Research development: A corresponding Author's countries analysis

**Fig. 3:**
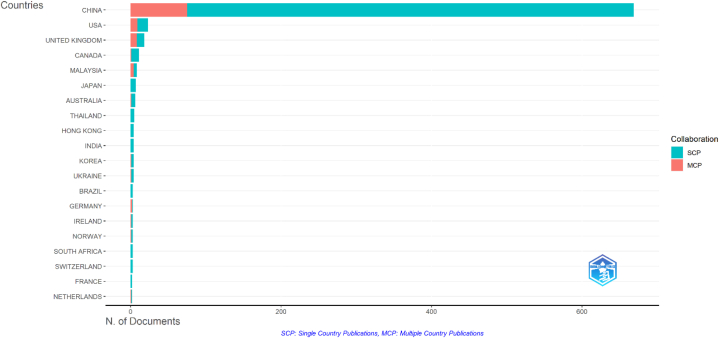
Total publications, SCP, and MCP of the top 20 countries.

To support the second research question regarding the development of bank lending and technological innovation research in excess liquidity countries, this research analyzes the most relevant countries by corresponding authors. Fig. 3 and Table 3 explain the distribution contribution of 25 out of 39 countries participating in this research on bank lending and technological innovation. The data reveals that China, USA, United Kingdom, Canada, Malaysia, Japan, Australia, Thailand, Hong Kong, and India are the leading countries in terms of research production. The results of this analysis show that research related to the relationship between the banking sector and technological innovation is not only exclusively carried out by researchers from countries that have high liquidity, such as Canada, China, Hong Kong, Japan, and Luxembourg, but is also carried out by researchers from countries other than those mentioned above detected to have high banking liquidity.

**Table 3:** Research development of the top 20 most relevant countries.

Country	Articles	SCP	MCP	Frequency	MCP_Ratio
China	669	594	75	0.712	0.112
USA	23	14	9	0.024	0.391
United Kingdom	18	10	8	0.019	0.444
Canada	11	10	1	0.012	0.091
Malaysia	8	4	4	0.009	0.5
Japan	7	7	0	0.007	0
Australia	6	5	1	0.006	0.167
Thailand	5	5	0	0.005	0
Hong Kong	4	4	0	0.004	0
India	4	4	0	0.004	0
Korea	4	3	1	0.004	0.25
Ukraine	4	3	1	0.004	0.25
Brazil	3	3	0	0.003	0
Germany	3	1	2	0.003	0.667
Ireland	3	2	1	0.003	0.333
Norway	3	2	1	0.003	0.333
South Africa	3	3	0	0.003	0
Switzerland	3	3	0	0.003	0
France	2	2	0	0.002	0
Netherlands	2	1	1	0.002	0.5

China dominates research with a contribution of 71.25 percent of the total scientific publications related to the relationship between the banking sector and technological innovation. Apart from being one of the top 5 most liquid banking countries, China's performance in supporting massive research in this field is also supported by technological developments in the financial and banking sectors. Pan et al. [28] explain that the extensive adoption of mobile payment platforms like WeChat Pay and Alipay facilitates the effective utilization of capital by individuals and small and medium enterprises (SMEs). In addition, digital inclusive finance enables inexpensive financing services to small and medium enterprises (SMEs) and individuals yet to obtain formal banking funding.

Fig. 3 and Table 3 also yield single-country publications (SCP) and multiple-country publications (MCP). The findings of this analysis indicate that research on bank lending and technological innovation is mainly characterized by collaboration among authors from a single country. This analysis shows that research related to bank lending and technological innovation is dominated by collaboration between authors from a single country, where SCP dominates MCP with a percentage of 88.07 and 11.93 percent, respectively. Conversely, Singapore, Egypt, Poland, and Saudi Arabia exhibit a complete author collaboration with authors from other countries, reaching 100 percent.

### 3.3 Research current focus: A trending topics analysis

This study provides document analysis based on trending topics to answer the second question of the current focus of bank lending and technological innovation research. The analysis employs the author's keywords along the timespan of investigation.

**Fig. 4:**
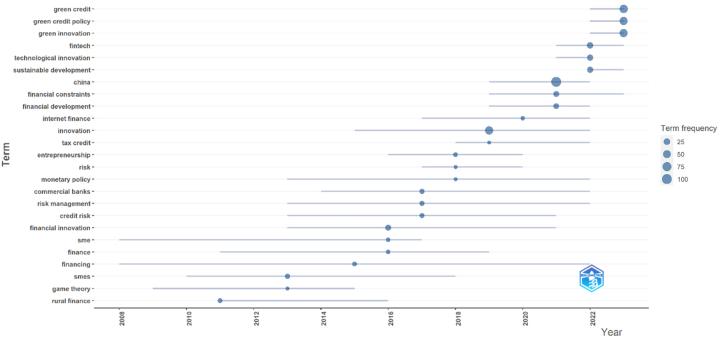
Research trending topics.

The parameters installed include a word minimum frequency of 5 and several words per year of 3. The result in Fig. 4 and Table 4 illustrates the progression of topics from 2011. Based on the frequency of keyword occurrences, the result shows that the 2007–2016 research topics are concentrated *on rural finance, game theory, SMEs, financing, finance, SME,* and *financial innovation* with relatively small frequencies. In 2017, the research moved to *credit risk, risk management,* and *commercial banks*, which had frequencies of 8, 9, and 10, respectively. *China* was the most elaborated topic in 2021, with a frequency of 104. The latest investigation concentrated on *green innovation, green credit policy,* and *green credit* with frequencies of 53, 54, and 58, respectively. *Green credit* has gained popularity in banking and technological innovation research since it refers to the loan capital banks or financial organizations provide to initiatives involved in conserving the environment and reducing energy consumption [51].

**Table 4:** Research topic development.

Item	Frequency	Year (Q1)	Year (Med.)	Year (Q3)
Rural finance	7	2011	2011	2016
SMEs	11	2010	2013	2018
Game theory	5	2009	2013	2015
Financing	7	2008	2015	2022
Financial innovation	16	2013	2016	2021
Finance	6	2011	2016	2019
SME	6	2008	2016	2017
Commercial banks	11	2014	2017	2022
Risk management	9	2013	2017	2022
Credit risk	8	2013	2017	2021
Entrepreneurship	7	2016	2018	2020
Monetary policy	5	2013	2018	2022
Risk	5	2017	2018	2020
Innovation	59	2015	2019	2022
Tax credit	5	2018	2019	2022
Internet finance	6	2017	2020	2022
China	104	2019	2021	2022
Financial constraints	17	2019	2021	2023
Financial development	15	2019	2021	2022
Fintech	23	2021	2022	2023
Technological innovation	22	2021	2022	2022
Sustainable development	21	2022	2022	2023
Green credit	58	2022	2023	2023
Green credit policy	54	2022	2023	2023
Green innovation	53	2022	2023	2023

### 3.4 Research thematic evolution: A conceptual structure analysis

**Fig. 5:**
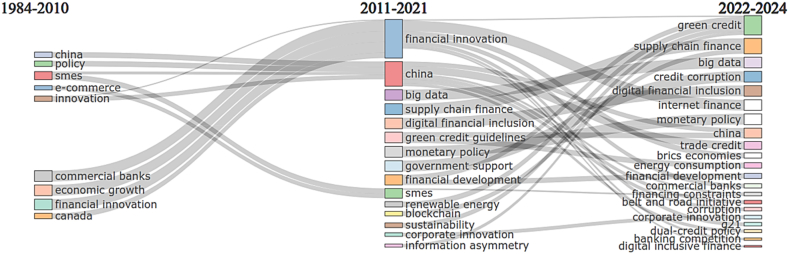
Research thematic evolution.

In response to the third inquiry of how the research theme evolution of bank lending and technological innovation in the top 5 liquid banking countries before and after the global financial crisis of 2008/2009 and during the COVID-19 pandemic, this study examines the vocabulary of documents to visualize the progression of thematic evolution dynamics. Firstly, this study decides how many cutting points divide the time slice along the span. Secondly, the analysis utilizes the term occurrence of keywords provided by the researchers to depict the evolutionary progression of topics on the bar chart shown in Fig. 5.

The study focuses on bank lending and technological innovation, significantly transforming environmental issues. Fig. 5 and Appendix 1 demonstrate that before and during the global financial crisis, research was concentrated on several traditional analyses, such as *commercial banks*, which focused on the early warning system and financial stability that mainly adjusted and echoed in the period of the global financial crisis [52]. Besides, research also focused on the macro interaction analysis of the role of banking intermediaries, which clustered in the *economic growth* theme [53,54]. Regarding the introduction of technological advancement to the financial sector, research started focusing on *financial innovation* during this period [55]. Moreover, this analysis found China and Canada to be the two most frequent regions.

After the global financial crisis and entering the outbreak of the COVID-19 pandemic, the three dominant research focuses shifted toward *financial innovation* [[56], [57], [58]] as the predominant theme from 2011 to 2021. Research focused on this theme discusses the role of technological implementation in supporting financial performance efficiency, resulting in improved economic growth.

Research themes have evolved after the COVID-19 pandemic (2022–2024) and supported the scientific development analysis. Since the exacerbation of carbon emissions, the world's climate has been adversely affected, and the environmental quality has been significantly harmed; research in bank lending and technological innovation focuses on diminishing this issue [48]. The banking sector is included in this agenda to serve as a crucial source of capital for sustainable project funding. Additionally, it helps address businesses' financial challenges when participating in green innovative financing by providing appropriate market instruments. Moreover, BRICS economies and G21 are founded as the most frequent regions. It consistently supports the previous finding that research in bank lending and technological innovation is not only implemented in countries with surplus banking liquidity but also broader agreements: BRICS and G21.

## 4 Discussion

Since its initial identification in 1984, there has been a rise in published studies focusing on quality and production investigations up to 2022. The increase in publications suggests that the fields of bank lending and technology innovation interest researchers. Research on the correlation between these two sectors typically shows an increasing trend in quality from 1984 to 2022 despite fluctuations in the number of citations. The research focuses on how financial institutions impact climate change and reduce environmental harm. The rise in citations is associated with the growth of research areas concerning banking and technology advancements [42,45,46,48,49]. It also implies that interdisciplinary development combines other areas, such as currency development and environmental engagement. This development matters in producing and citing research in bank lending and technological innovation.

Most relevant national analyses emphasize progress in technological innovation and research on bank lending. The analysis indicates that countries with robust banking liquidity are not the sole focus of banking and technical innovation research. New Zealand ranks among the top 20 countries in research in this field. However, research on this topic is also increasing and being carried out by researchers in the USA, UK, Malaysia, and other nations.

Analysis of trending topics reveals that research in this field goes beyond banking and its influence on innovation. Since 2008, scholarly discourse has centered on the significance of financial development, particularly on funding, with frequencies generally staying below 10. The emergence of new technologies has redirected research emphasis in this field towards internet finance, which garnered considerable interest in early 2017 and fintech in 2021. Contemporary research in this area focuses on green innovation, green financing, and green lending policies. The current research broadly examines the involvement of banks and financial institutions in supporting the green economy movement.

Moreover, theme evolution analysis also supports comparable results. Analysis of thematic evolution research classified into three areas shows significant improvement. Between 1984 and 2010 (before the financial crisis), research focused on commercial banking, economic growth, and technical innovation. Most research focuses on the impact of the financial crisis on banking system development and recovery, as revealed by Kuang & Zhou [54], C. Li [55], Sun et al. [52], and Xiong et al. [53].

The three themes evolved into themes related to financial innovation between 2011 and 2021, when the financial crisis was over and entered the period of the COVID-19 pandemic. Research on the green economy began that year, specifically on renewable energy and sustainability. Besides, the introduction of financial technology (fintech) pushes the banking industry to enhance its services and operational innovation to gain sustainable competitive advantage [56]. C. C. Lee et al. [57] explain that banks engaged in fintech have the lowest cost efﬁciency and operate under inferior technology. Therefore, it positively impacts credit and economic growth [58].

Between 2022 and 2024 (post-pandemic COVID-19), research has shifted towards renewable energy, sustainability, bank lending, and technological innovation, with a particular emphasis on green credit, as discussed in the study by Irfan et al. [48]. The study results are consistent with the overview and analysis of current themes. Due to this emerging issue, *green credit* has gained popularity in banking and technological innovation research since it refers to the modern loan capital banks or financial organizations provide to initiatives involved in conserving the environment and reducing energy consumption. This result is consistent with the document analysis, which provides green credit as the most trending topic in bank lending and technological innovation research.

Research on bank lending and technological innovation has significantly advanced. The analysis findings demonstrate progress in research on bank lending and technological innovation in terms of production volume, citation quality, and detailed themes. This is evidenced by fundamental overview analysis, country-specific analysis, trending topic analysis, and thematic evolution.

## 5 Research elevation and further direction

**Fig. 6:**
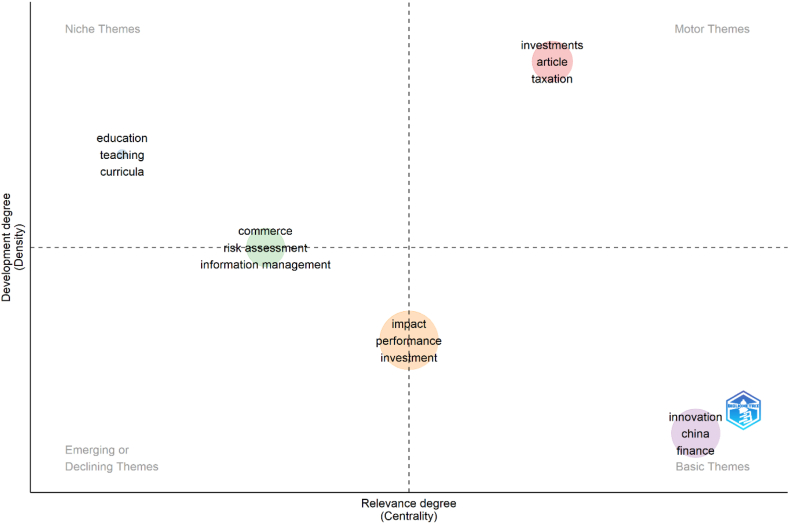
Research thematic map.

To provide further research directions, this study employs a thematic map in two dimensions—density and centrality—defined by Callon et al. [40] using keyword co-occurrences. According to Cobo et al. [37], density indicates a network's internal strength, whereas centrality indicates how much a network interacts with other networks or how relevant it is. A study area can be represented in both metrics as a collection of research themes based on their clustering. These themes are then projected onto a two-dimensional framework and categorized into four quadrants, as illustrated in Fig. 6.

• The notion of *investment* is a prominent theme inside the cluster network, with a high centrality score of 4.584 and a density score of 26.620. It indicates that the theme is established and is vital in arranging the research topic.
• The cluster labeled as *education* has been identified as a specialized theme. This theme has a high density of 37.083 and a low centrality of 0.165, indicating minimal relevancy.
• The *innovation* is recognized as a basic theme with a high level of centrality (7.193) and a low-density level (16.897). This theme is fundamental and significant but needs more thorough development.
• The term *commerce* is positioned between the niche and emerging quadrants. It has a higher centrality (4.776) to *education* and a higher density (42.876) to *impact.* This theme needs to be more sparsely elaborated and peripheral.
• The *impact* is positioned within the quadrant between the primary and emerging themes due to its lower centrality value of 2.591 and more considerable density value of 32.765 about *innovation.* Thus, this subject receives limited attention and is of secondary importance in bank lending and technical innovation studies.

This study offers various avenues for further research based on the thematic classification map in Fig. 6 and Appendix 2. Initially, investment is the subject that stands out the most in popularity, density, and centrality. This theme can catalyze combining with other themes requiring further development. Research with keywords such as *government taxation* [47], *credit provision* [26], *sustainable development* [59], and *emission control* [60] have high occurrences and significant attention from researchers.

The notion of *education* necessitates future elevation. The low-relevance degree needs the specialty theme integrated with the motor category. The detected research [61,62] provides keywords *curricula, engineering education, students,* and *teaching* that have low frequencies. The research in this cluster analyzed the implementation of technological innovation in education operational systems, such as online platforms [61] and technological entrepreneurship [62]. To elevate the engagement of this cluster, detected keywords can be examined alongside the terms of *emission control, sustainable development, taxation, and articles* from the *investments* cluster, which have high occurrences.

Furthermore, within a basic cluster, innovation plays a crucial role in the research of banking and technological innovation of economies with surplus liquid assets. This cluster has high collaboration with other themes. Therefore, it developed more and attracted high citations when studying bank lending, technological innovation, environmental issues [63], and digital financing [28]. The thematic map analysis shows high occurrences of keywords such as *pollution, subsidy and sustainability, cost and debt, banking, credit constraints, financial development, industry, competition, policy, finance,* and *China*. Nevertheless, it is necessary to elevate the lowest density. Further investigation is necessary to internally elevate the understanding of topics with limited instances, such as *corporate innovation, credit risk, energy efficiency, globalization, liberalization, product development,* and *returns.*

Cluster of commerce is dominated by research in commercial banking and risk management [64,65]. Furthermore, elevating the centrality of this cluster is necessary. It implies that this cluster needs the internal and external fortitude derived by collaborating with other networks. The keywords such as *agriculture, credit ratings, environmental technology, financial institution, laws and legislation,* and *learning systems* with low occurrences can be enhanced internally and by incorporating additional keywords from the investment cluster.

Ultimately, the *impact* cluster is dominated by research on financial constraints and environmental issues [42,44], which attracted significant research attention. However, this cluster should also be enhanced regarding centrality and density. It implies that this cluster requires the external and internal strength of the network. The infrequently used keywords, such as *agglomeration, antecedents, construction, demand, development investments, drivers,* and *electric vehicles,* require further internal investigation and collaboration with the *investment* cluster. Consequently, these clusters can be relocated to the upper quadrant.

## 6 Conclusion

The empirical debate between the role and lack of contribution of bank financing and technological innovation activities left inconclusive record. Earlier group of studies revealed a favorable connection between bank lending and technological innovation. However, other group of scholars found an inverse result. In this inconclusion debate, studies evaluate the research evolution and elevation to guide further potential analysis in this area is also rare. Therefore, to address this issue, this study examines the production, development, topics, and thematic evolution in bank lending and technological innovation and suggestions for further research elevation with unique settings. First, this study employs bibliometrics analysis to observe the production, quality, topic and theme movement, and thematic map analysis that helps to answer the research questions. Second, we employ specific keyword countries with excess banking liquidity to provide closer correlation between bank lending and technological innovation.

Our analysis provides significant developments in performance and quality, corresponding, topic, and theme, as follows.

1. Scientific performance and quality: the bibliometric analysis reveals significant advancements of bank lending and technological innovation research with focus on countries with excess liquidity since its inception in 1984. The overall data indicate that the number of published papers is increasing with their citations.
2. Country's corresponding research: the study found that research samples not only from specific countries with excess liquidity, but also broader integrated nations such as BRICS and G21 to investigate the relationship between the banking sector and technological innovation.
3. Trending topics and thematic evolution: analysis of topic development reveals the predominant environmental and economic challenges. The thematic evolution results also support the research development that dominated by green credit theme.
4. Research thematic map: research in cluster of investment can help to improve other detected themes such as education, innovation, commerce, and impact. Therefore, research in bank lending and technological innovation would be more elevated.

This study enhances our comprehension of the ongoing intellectual discourse in bank lending and technological innovation research. It also concludes that there is a significant and quick expansion of research in this area, particularly in the most financially stable countries. The investigation has yielded valuable data for researchers, practitioners, and government institutions in countries with surplus liquidity. Additionally, the visualized map has offered insightful information and extensive comprehension of key institutions, organizational and national partnerships, the current state of the research field, and the development trend. This research offers an overview of how the role of finance has evolved for regulators and bankers to enhance financing and liquidity, particularly for innovation and environmental purposes. From a practical standpoint, various stakeholders would benefit from comprehending the influence of bank lending and technology innovation on social, financial, regional, and ecological improvements. First, government agencies and business organizations who are deciding upon policy-making, consulting, and research cooperation. Second, students who begin to assess the state of research gaps and current popular theme in the finance-innovation field. Third, when academicians identify concentrated research in the finance-innovation field in countries with excess liquidity or need to find possible broader collaboration with other academicians.

Nevertheless, the present investigation has several limitations. Material is exclusively gathered from Scopus and WOS libraries, which currently need to be improved in covering all the published literature on bank lending and technological innovation. Results may differ if data is collected from alternative resources for varying periods. It is a limitation of bibliometric analysis, which may have a small impact on the analysis from a broad perspective. This constraint may also arise during the data cleansing process. The association between each document and bank lending and technological innovation was identified manually to the greatest extent possible. Errors in the marginal literature during the identification phase may have occurred, although they had little impact on the overall analysis. We have studied the co-occurrence of authors' keywords in scientific mapping. Further aspects of science mapping may be addressed.

## Data availability

Data will be available on request.

## CRediT authorship contribution statement

**Agus Salim:** Writing – review & editing, Writing – original draft, Methodology, Investigation, Formal analysis, Data curation, Conceptualization. **Suripto:** Visualization, Supervision, Investigation, Data curation, Visualization, Supervision, Investigation, Data curation. **Dini Yuniarti:** Visualization, Supervision, Project administration, Data curation. **Ignatius Abasimi:** Methodology, Investigation. **Nurul Azizah Az Zakiyyah:** Software, Project administration, Investigation. **Indanazulfa Qurrota A'yun:** Visualization, Formal analysis.

## Declaration of competing interest

The authors declare that they have no known competing financial interests or personal relationships that could have appeared to influence the work reported in this paper.

## Figures and Tables

**Table:** 

From		To		Words	Weighted Inclusion Index	Inclusion Index	Occ.	Stability Index
1984–2010	Canada	2011–2021	Financial innovation	Sustainable development	0.50	0.50	2	0.03
	China		China	China	0.50	0.50	2	0.02
	Commercial banks		Financial innovation	Commercial banks	1.00	1.00	2	0.03
	e-commerce		SMEs	Game theory	0.40	0.50	2	0.14
	Economic growth		Financial innovation	Economic growth	1.00	1.00	2	0.03
	Financial innovation		Financial innovation	Financial innovation	1.00	1.00	2	0.03
	Innovation		China	Innovation	0.40	0.14	8	0.02
	Innovation		Financial innovation	Taiwan	0.10	0.14	2	0.03
	Policy		China	SME	0.50	0.50	2	0.02
	SMEs		China	Finance	0.25	0.33	2	0.02
	SMEs		SMEs	SMEs	0.50	0.33	4	0.13
2011–2021	Big data	2022–2024	Big data	Big data	1.00	1.00	3	0.25
	Blockchain		Supply chain finance	Blockchain	0.43	0.50	5	0.14
	China		Belt and Road Initiative	Political connection	0.33	0.33	2	0.02
	China		China	China; innovation; green credit policy; R&D investment; porter hypothesis	0.43	0.04	51	0.01
	China		Corruption	Tax credit	0.33	0.33	2	0.02
	China		Digital finance	Digital finance	0.05	0.10	2	0.02
	China		Dual-credit policy	Dual-credit policy	0.29	0.50	2	0.02
	China		Financing constraints	Financial constraints; financing constraints	0.20	0.10	10	0.02
	China		Green credit	Green innovation; difference-in-difference model	0.04	0.02	4	0.01
	China		Trade credit	Trade credit	0.71	0.50	5	0.02
	Corporate innovation		Corporate innovation	Corporate innovation	0.33	0.33	2	0.14
	Credit risk		Green credit	Credit risk	0.07	0.08	6	0.02
	Digital financial inclusion		Digital financial inclusion	Digital financial inclusion	1.00	1.00	2	0.33
	Financial development		China	Environmental regulation	0.06	0.07	3	0.03
	Financial development		Financial development	Financial development; industrial structure	0.47	0.20	9	0.06
	Financial development		Green credit	Green credit; technological innovation; green finance; environmental pollution; mediating effect	0.47	0.07	7	0.02
	Financial innovation		Banking competition	Financial innovation	0.22	0.33	12	0.03
	Financial innovation		BRICS economies	Economic growth	0.50	0.50	3	0.03
	Financial innovation		China	Sustainable development	0.03	0.04	3	0.02
	Financial innovation		Commercial banks	Commercial banks	0.40	0.25	5	0.03
	Financial innovation		Digital finance	Financial inclusion	0.05	0.10	2	0.03
	Financial innovation		Digital inclusive finance	Financial regulation	0.15	0.20	2	0.03
	Financial innovation		G21	Shadow banking	0.33	0.33	4	0.03
	Financial innovation		Green credit	Fintech; risk management	0.10	0.03	7	0.01
	Financial innovation		Internet finance	Internet finance	1.00	1.00	4	0.03
	Government support		Credit corruption	Government support	1.00	1.00	2	0.25
	Green credit guidelines		China	Green credit guidelines	0.50	0.50	2	0.04
	Green credit guidelines		Energy consumption	Heterogeneity	0.50	0.50	2	0.17
	Information asymmetry		Green credit	Information asymmetry	0.30	0.25	3	0.02
	Monetary policy		Monetary policy	Monetary policy	1.00	1.00	2	1.00
	Renewable energy		Green credit	Renewable energy	0.50	0.50	2	0.02
	SMEs		Financing constraints	SMEs	0.13	0.17	5	0.07
	Supply chain finance		Supply chain finance	Supply chain finance	1.00	1.00	4	0.17
	Sustainability		Green credit	Sustainability	0.43	0.33	3	0.02

**Table:** 

Cluster	Centrality	Density	Rank Centrality	Rank Density	Cluster Frequency	Occurrence and Keywords
Commerce	4.776	42.876	4	5	330	(4) agriculture, blockchain, credit ratings, environmental technology, financial institution, green credits, industrial economics, information science, laws and legislation, learning systems, and risk controls; (5) economic analysis, financial market, financial system, game theory, research, small and medium-sized enterprise, and supply chains; (6) credit risks, economic growths, information technology, international trade, optimization, and risk management; (7) economic and social effects, electronic commerce, and technological innovation; (8) commercial bank, financial crisis, and regression analysis; (9) financial innovation and financial service; (10) big data; (16) industrial engineering; (17) economics; (18) information management; (20) commerce and risk management.
Education	0.165	37.083	1	4	22	(4) curricula, engineering education, students, and teaching; (6) education
Impact	2.591	32.765	2	3	279	(4) agglomeration, antecedents, construction, demand, development investments, drivers, electric vehicles, environmental innovation, financial performance, income, industries, investment evidence, liquidity, matter, prediction, and slacks-based measure; (5) air-pollution, behavior, corporate, corporate social-responsibility, decisions, development investment, distance, foreign direct-investment, framework, impacts, mechanism, networks, nexus, political connections, prices, public subsidies, spillovers, and subsidies; (6) business, capabilities, carbon emissions, dependence, dynamics, firm performance, institutions, panel, policies, responsibility, and trust; (7) adoption, firm innovation, intensity, perspective, and strategies; (8) access, energy-consumption, enterprises, environmental-regulations, performance evidence, productivity growth, size, strategy, system, systems, and technological-innovation; (9) absorptive-capacity, companies, disclosure, empirical-evidence, panel-data, property-rights, renewable energy investment, and transition; (10) cash flow, corporate governance, cost, economy, equity, and regulations; (11) emissions, incentives, and renewable energy; (12) development studies and uncertainty; (13) entrepreneurship, financial constraints, and firm; (14) consumption and trade credit; (15) eco-innovation, empirical-analysis, markets, and model; (16) quality; (17) market; (18) banks, energy, and trade; (19) governance; (22) CO2 emission and ownership; (23) determinants; (25) credit policy and risk; (28) productivity; (30) management; (31) information and technology; (36) efficiency; (38) firms; (47) economic-growth; (48) constraints; (51) research-and-development; (66) credit; (80) growth; (94) investment; (96) performance; (112) impact.
Innovation	7.193	16.897	5	1	692	(4) corporate innovation, credit risk, energy efficiency, globalization, liberalization, product development, and returns; (5) emerging markets, microfinance, and SMEs; (6) cities, corruption, internet, and probability; (7) agency costs, capital structure, environment, environmental performance, financing constraints, and urbanization; (9) models; (10) pollution; (11) subsidy and sustainability; (12) cost and debt; (15) banking; (18) credit constraints; (20) financial development; (26) industry; (33) competition; (50) policy; (66) finance; (98) China; (192) innovation.
Investments	4.584	26.620	3	2	1699	(4) climate change, commercial phenomena, developing countries, developing world, empirical analysis, employment, environmental impact, Europe, health, innovation performance, invention, organization and management, organizations, subsidy system, support, tax credits, and taxes Investments; (5) inventions, North America, organization, and technological development; (6) incentive; (7) carbon emission and Japan; (8) economic development, environmental management, environmental protection, green economy, and United States; (9) emission control, environmental economics, and humans; (10) carbon, credit provision, environmental policy, public policy, and research and development; (12) human; (13) Canada, government, and sustainable development; (15) taxation; (19) article; (20) investments
